# Public and Private Nursing Institutions

**Published:** 1887-04-09

**Authors:** 


### Public and Private Nursing Institutions.

By Mrs. Bedingfeld, of the Belgravia Institute for Nurses.

Though unwilling to trespass further on your space, may I say a few words regarding Miss Manson's letter in your issue of March 19th? I regret that Miss Manson has taken the remarks made on her article in such an adverse spirit. She is but one among a number, and whilst enjoying the courage of her own opinions, must allow others to do the same. I did not say that it was "incorrect" that private nursing institutions do, to use again Miss Manson's own expression, "farm out nurses for their own profit" (by the way, "Nurse-farming" is not a very elegant term! Does Miss Manson class us with "Baby-farmers"?), but implied, and still do so, that it is unfair to censure them for doing the very thing the hospitals are about to do; especially as institutions (I am speaking of strictly private ones) do not appeal to the public for subscriptions, nor are the superintendents salaried officers, as hospital matrons are; therefore, *if* any profit is made at the end of a number of years of anxious work, surely the one who has had to bear the brunt of *all* the expense, *whether the institution is a success or not*, has a right to it. Institutions which do not make public appeals to charity, are not bound to give an account of their takings, simply to gratify curiosity; but I will say, for my own part, that my experience is similar to that of "A Country Lady Superintendent," viz., that after rent, taxes, wear and tear of furniture, nurses' salaries, board, and sundries are paid, there is but little surplus. If there is any it has to be put by to meet any deficit during unsuccessful years. Miss Manson in what she says about some institutions taking untrained nurses, commits the same fault that she lays to my charge, viz., imperfect quotation; and I maintain what I said, that institutions take them as "probationers" only, sending them to be trained for the proper course. The cases quoted by Miss Manson seem to be those of women who have entered the hospitals *on their own account*, and *not* sent in by institutions; and if some nurses are content with as little as six months' training (I have but rarely come across these, and refuse to employ them when I do), the entire blame and responsibility rest with the hospitals who admit them for this short time. If the hospitals would refuse to train anyone for less than a year, or whatever time longer they like to fix, the fault would cease; but so long as they, for the sake of the *£ s. d.*, admit women on payment of a guinea a week for a limited and short period, so long will women be found to avail themselves of the opportunity. Ladies often send their maids to acquire this superficial smattering, and so save themselves in illness the expense of a "trained nurse." I am quite as much alive as Miss Manson herself to the desirability of a three years' training, but all who have not had so long must not be utterly condemned, as some women will learn more in a year than others will in three or four. I am quite within the truth when I say that good nurses have been almost starving this winter, even though parish and district nurses are in such demand. These latter are, for reasons I will give, most difficult to get; for here again is a nurse's education not complete when she leaves the hospital, in which she has every modern appliance to use. She has, before she is fit for district work, to learn, for one thing, to make the best of the materials at hand. Then a district nurse is often required to be a qualified midwife, in fact, more stress is frequently laid upon this than upon her medical and surgical skill. And a district nurse must have extra good health, as she must be out in all weathers; and be gifted with exceptional tact and love of her work, as she has many disagreeables to put up with; and the poor are *very* sensitive, and require to be very carefully dealt with. (I speak this from my own personal experience as midwife among the poorer classes for over eight years.) Lastly, the salary offered to parish nurses and midwives is so inadequate, the maximum average sum being one pound a week, out of which they have to find board, lodging, firing, clothing, and all incidental expenses, that few care to undertake the work. Pardon this digression. The subject is an important one. Everyone with the most rudimentary knowledge of nursing knows that *washing dresses* are a necessity for a nurse. The objection was to "indoor and outdoor uniform." And I repeat that in many illnesses patients object to be perpetually reminded by the nurse's attire that they are invalids. And the variety in colour and form of the dresses worn by nurses who are not bound to wear uniform, is often a relief to the monotony of the sick-room. Superintendents should see that the nurses employed by them have suitable dresses, but in my opinion compulsory uniform is not desirable, except in hospitals and infirmaries. Miss Manson fails to answer one of my questions, and a very important one, viz.: when private nursing institutions are swept away, and *all* hospitals, country as well as town ones, have raised the funds to build the necessary homes for their private nurses, and the superlative nurses are trained to occupy them, what is to become of those hundreds who have been, and are now annually sent out from the various hospitals certified as efficiently trained, and who, though now nursing either from existing institutions or on their own hands (and this latter is a large class forgotten by Miss Manson), will then be left without the means of earning their own livings? Will the hospitals who have trained them receive them back, or pension them? If not, what is to become of them?

Miss Manson doubtless considers no nurses equal to those *she* has trained. But this is no reason for crying down those who, though *not* trained at St. Bartholomew's, are doing good work outside hospital walls. I have had several letters thanking me for mine in your issue of March 5th, and I am pleased to see that Miss Wilson, of whose large and varied experience in nursing one cannot speak too highly, and whose opinion one is bound to respect, agrees in the main with me, and to find that my opinions are not altogether isolated ones.

I should like to say that the above was written and in type before the appearance of The Hospital last week, which must be my apology for the seeming repetition of some of the opinions advanced by others in that number.

By a Provincial Hospital Matron.

As the subject of nursing institutions in connection with hospitals has long interested me, perhaps I may be allowed to add a few words to the discussion which has been carried on through your paper. I fully agree with Miss Manson in thinking that it is natural and right that leading hospitals should have nursing institutions attached to them. It does not seem to me that this will involve an attempt to "Serve God and Mammon"; the aim of the hospital and the home would be one, namely, to provide the sick with skilled attendants. Whether the sick are possessed of this world's goods or not, is surely a side question. I fail to see that Miss Manson's opponents have answered the charges (if I may use the expression) brought by her against many nursing institutions, or have shown *why* hospitals should not send out private nurses. Miss Manson says that "many institutions send probationers for six months or a years' training to some hospital, and then send them out as trained nurses." This is perfectly true, of some provincial institutions at any rate, and the evils of such a system are very great. In the first place, no woman can possibly be called "trained" at the end of six months, and no hospital that I ever heard of will give her a certificate. Mrs. Bedingfeld throws the responsibility of short terms of training on to the hospital; but surely, if the committee of an institution say to the committee of a hospital, "We want our probationers trained, but we cannot afford to spend £25 on each probationer, so we will send them to you for a term of six months only," the hospital, by refusing a certificate to such probationers, throws the responsibility on to the institution; for the public have to take the word of the institution, and the institution only, for the proficiency of the women sent to them as nurses.

But even if a year's training be allowed, is that enough? Some women are slower to learn than others; the slow one may be a valuable nurse in the end, but she requires time, and this can be given her if she is attached to a hospital, but hardly if she belongs to an institution. Then, if an institution be badly off for funds, the committee is tempted to choose a hospital, not because the training is good, but because, having a somewhat insufficient staff (numerically), it is only too glad to get extra workers, not only without expense, but for a small payment. It is not long since I read the report of an annual meeting of an institution in which a member of the committee referred with pleasure to the fact that they were getting their probationers trained at half-price. The quality of the training was not commented on.

There is another difficulty in the way of the training-out system. All superintendents of nursing know that a woman may be considered "promising," so long as only probationer's work is expected of her, and may yet be found wanting as she advances in her nursing, and has to use her brains. Will the committee of the institution be willing to withdraw her, and forfeit the five or six pounds already paid for her training? or will the hospital always be willing to bear the losses incurred by unsatisfactory probationers? I know in some cases probationers' deficiencies have been passed over for these reasons.

It must be more possible for a hospital matron who has been a nurse, in her daily work to judge of her capacities than it is for the most efficient superintendent of a Home, who only sees a nurse in her hours of leisure. A "Private Nurse" says that in hospital "individuals pass uncriticised." If this is true of any hospital, it certainly is not one which can be called a training school, whatever other good work it may accomplish.

The other objection, that nurses straight from hospital do not understand the "niceties and refinements" of the sick-room, may surely be answered by saying that that depends entirely upon the individual nurse, and the class from which she is drawn—a maid-of-all-work turned into a nurse probably would not.

In conclusion, I may say that the question at stake is this: How may we best insure that the public shall be supplied with efficient nurses? This question can only be satisfactorily answered by hearty co-operation between hospitals and nursing institutions. If the latter only send out as "trained nurses" those who have satisfactorily completed a full term of training (by which I mean that they should have been tested for some part of the time as staff nurses), I do not see why they should not retain the confidence of the public; but this discussion will do good if it drags to the light those who, for the sake of satisfying the demands of the public, or for maintaining a respectable balance at the bankers, send out inexperienced women as skilled workers, and thereby lower the profession of nursing, and prejudice the public (many of whom do not understand that there is training, and training) against those who have honestly tried to perfect themselves in the work of their calling. No system is perfect; but if the matrons of hospitals and nursing institutions would work together for a common end—the raising of the standard of nursing in all its branches—many of the evils now complained of would be avoided.

